# Nanopillar array with a λ/11 diameter fabricated by a kind of visible CW laser direct lithography system

**DOI:** 10.1186/1556-276X-8-280

**Published:** 2013-06-11

**Authors:** Chen Zhang, Kaige Wang, Jintao Bai, Shuang Wang, Wei Zhao, Fang Yang, Changzhi Gu, Guiren Wang

**Affiliations:** 1 Institute of Photonics and Photo-technology, International Scientific and Technological Cooperation Base of Photoelectric Technology and Functional Materials and Application, Northwest University, 229 North Taibai Rd, Xi'an, 710069, People's Republic of China; 2 Department of Mechanical Engineering, University of South Carolina, 300 Main St., Columbia, SC, 29208, USA; 3 Institute of Physics, Chinese Academy of Sciences, 8 Zhongguancun 3rd South St, Beijing, 100190, People's Republic of China; 4 Department of Physics, Northwest University, 229 North Taibai Rd, Xi'an, 710069, People's Republic of China; 5 Department of Biomedical Engineering Program, University of South Carolina, 300 Main St., Columbia, SC, 29208, USA

**Keywords:** Nanopillar, Diffraction limit, Lithography, Coma, Astigmatism

## Abstract

Nanoscale functional structures are indispensable elements in many fields of modern science. In this paper, nanopillar array with a pillar diameter far smaller than Abbe's diffraction limit is realized by a new kind of continuous wave (CW) laser direct lithography technology. With atomic force microscopy technology, the average diameter of nanopillars on thin OIR906 photoresist film is about 65 nm and the smallest diameter is 48 nm, which is about 1/11 of the incident laser wavelength. Also, the influences of coma and astigmatism effects to the shape and size of nanopillar are numerically simulated by utilizing vector integral. As far as we know, it is the first time that nanopillar array is implemented by a donut-shaped 532-nm visible CW laser. The study presents a new, simple, inexpensive, and effective approach for nanopillar/pore array fabrication.

## Background

Nowadays, nanoscale structures such as nanopillar and nanopore arrays are considered essential functional nanotexturizations for modern scientific research and application. Nanopillar arrays have been employed in the study of field emission [1], solar cell industry [2], biological sensing [3], micro-/nanoscale fluidics, near-field optics, and the lab-on-a-chip technology [4]. Nanopore arrays have also been recognized as valuable structures in many advanced fields such as photovoltaic [5] and photonic crystal research [6], gas detection [7], and especially in biological molecules detection and separation [8]. Fitting with foregoing scientific advancements, the nanoscale fabricating methods and technologies have been made good progress. Nanopillar and nanopore arrays can be fabricated with direct growth approaches (metal-organic chemical vapor deposition, hydride vapor phase epitaxy, molecular beam epitaxy) [9-11], nanosphere-assist etching [12,13], electronic beam lithography [14,15], nanoimprint technology [16], and laser lithography [17].

Since the merits of fabricating speediness and cleanliness, maskless process, controllable pattern shape and size, and capability of lithograph in three dimensions [18,19], laser direct lithography technology is one of the most attractive approaches to fabricate nanoscale functional structures as compared with the disadvantages such as expensive, heavy, or low precision of other methods. Choi's group has reported implementing 100-nm-level nanostructure arrays over a large scale by means of laser interference lithography [20-23]. Scott and Li have respectively fabricated sub-100-nm isotropic voxel [24] and voxel with a 40-nm axial size [25] by photo-initiation inhibiting technology. Cao has obtained a nanoline with a width of 130 nm and nanodots with a diameter of 40 nm [26] by polymerization inhibiting, too. In Andrew's work, the nanolines with an average width of 36 nm were drawn employing absorbance modulation lithography [27]. Tanaka and Thiel have shown fabricating spatial voxel to sub-120 nm with the two-photo-absorption technology [28,29]. Qi got a single polymerized tip with a diameter of 120 nm with the same technical route [30].

However, the utilization of femtosecond laser systems makes the lithography system complex and expensive. Even, in a continuous wave (CW) laser two-photon absorption method, photoresist is tailored and the whole system is costly. Furthermore, two laser sources are required in both photo-inhibiting and absorbance modulation methods, and the photoresist materials should have particular properties that result in restrictions in choosing light sources and resist materials.

In the paper, we will report a kind of nanopillar array with a pillar diameter much smaller than Abbe's diffraction limitation by visible CW laser direct lithography technology. A 532-nm CW laser beam, which is modified by a phase mask to generate a nanolevel dark core in the focus space, is proposed and applied in nanopillar/pore fabricating. The nanopillar array is obtained when the laser beam is irradiated to the positive tone photoresist, while nanopore will be generated with a negative tone photoresist. To the best of our knowledge, this is the first time that nanopillar arrays are fabricated with a spatial donut shape, structured visible CW laser. Experimental results are measured by AFM, and the distortion and the inconsistency of nanopatterns are analyzed with theoretical simulation. This preliminary work explores a novel, easy, and effective method of maskless CW laser direct writing technology to carry out functional nanopillar/pore arrays.

## Methods

**Figure 1:**
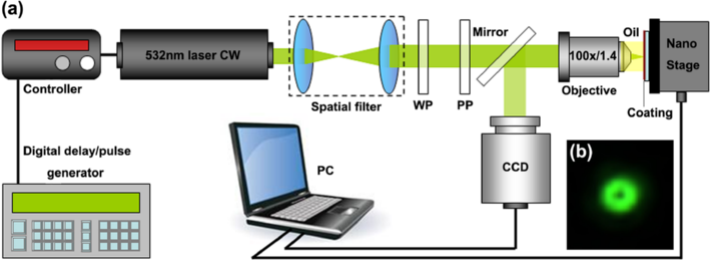
Schematic diagram of experimental setup (a) and laser focal spot (b).

The laser direct writing system in our experiments is schematically shown in Figure 1a. The light source is a CW laser with its center wavelength at 532 nm (DHOM-VL-532-2000, Suzhou Daheng Optics and Fine Mechanics Co., Ltd, Suzhou, China). A spatial filter is placed behind the laser head to achieve a high-quality beam mode. A λ/4 wave plate (WP) is used to transfer the linearly polarized 532-nm laser into a right-handed circularly polarized beam. A vortex phase plate (PP) changes phase from 0 to 2π in anticlockwise direction. Here, a high numerical aperture (NA) (1.4) oil-immersed objective (Apoplan 100×/1.4, Olympus Optical Co., Ltd, Tokyo, Japan) is employed to focus the laser beam. Laser power at the input pupil of the objective is approximately 16 μW. During laser lithography, the photoresist-coated glass wafer is mounted onto a three-dimensional (3D) piezoelectric scanning stage (P-611.3SF along with the E-664.S3 Amplifier/Controller, Physik Instrument, Auburn, MA, USA). The rapid motion of PI stage is controlled by a PC program. Laser was triggered by a digital pulse generator (DG535, Stanford Research System, Inc., Sunnyvale, CA, USA), and pulse lasting time is 120 ms. A high-performance digital charge-coupled device (CCD) camera (QICAM, QImaging Co., Ltd, Surrey, Canada) is applied for alignment and imaging. Figure 1b is the laser spot imaged in the focal plane by the CCD. This structure of laser beam has been utilized during the following nanopillar array fabrication. Positive tone photoresist (OIR906, Fujifilm Electronic Materials USA, Inc., Valhalla, NY, USA) is adopted through the whole experiment. This resist is coated on a glass wafer by a spinner, and its thickness is approximately 800 nm.

In principle, with the modulation of the vortex phase-shifting plate, the circularly polarized Gaussian beam is generated as a donut-shaped pattern on the focal plane. The dimension of the dark core of the donut-shaped pattern is smaller than the diffraction limitation [31]. During the experiment, the photoresist at the center of the pattern will not be exposed because of the null intensity point. Since the positive tone photoresist was applied in this work, a kind of nanopillar structure, whose diameter is far below diffraction limitation, could be obtained in the center of the donut-shaped pattern with appropriate input laser power.

The procedure of experiment is composed of the steps of spin coating, preexposure baking, exposing, post-exposure baking, developing, and hard baking in sequence. The obtained nanostructures are measured, characterized, and analyzed with an atomic force microscopy (AFM, Veeco Dimension 3100 AFM system, Veeco Instruments Inc., Plainview, NY, USA). To obtain the nanopatterns with high precision and consistency, the focal sphere should be accurately focused onto the surface of the photoresist. Furthermore, the motion of the scanning stage is required to be synchronized with laser exposure for fast fabricating nanopatterns.

## Results and discussion

### Experimental results

**Figure 2:**
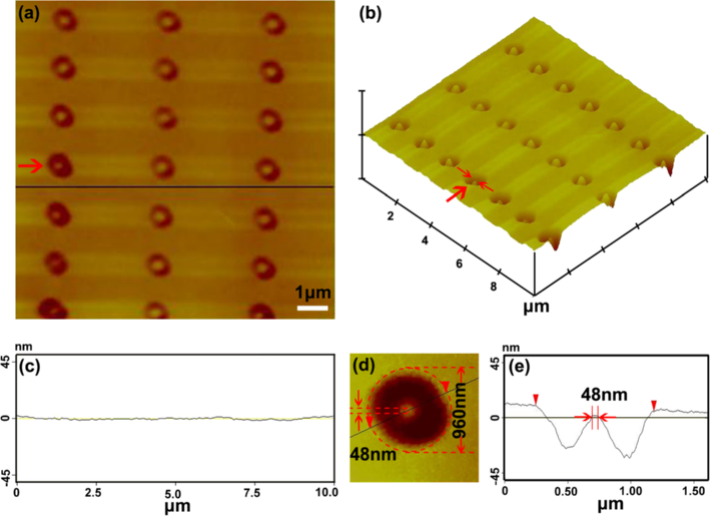
**Typical image of a nanopillar array fabricated in the experiments.** (**a**) AFM image of nanopillar array fabricated with 532-nm CW laser and (**b**) its corresponding 3D image. (**c**) Roughness of coating along the dark line in (**a**). (**d**) Enlargement of one unit and (**e**) its cross section marked in (**a**).

Figure 2 is a typical image of a nanopillar array fabricated in the experiments. The top surface pattern of the overall topography is displayed as Figure 2a. The scan range is about 10 μm × 10 μm. Each nanopillar is located in a circular pit whose external diameter is around 950 nm. The average diameter of the nanopillar is 65 nm, which is much smaller than the size of Abbe's limit. Figure 2b is an AFM 3D image of the nanopillar array. Figure 2c represents the cross-sectional topography along the dark line which is shown in Figure 2a, and it illustrates the flatness of the coating surface. Figure 2d, e shows more details about the typical nanopillar in the array. Figure 2d is the top view of the nanopillar which is marked by the arrow in the nanopillar array of Figure 2a. A dark line in Figure 2d acts as the symmetry axis of the pattern. It passes through the apex of the nanopillar, and its corresponding cross-sectional image is illustrated in Figure 2e. With careful calibration and analysis, it is found that the diameter of the pillar is around 48 nm, which is about λ/11, much smaller than the diffraction limit λ/2, where λ is the incident laser wavelength at about 532 nm. Figure 2 demonstrates that the nanopillar array can be manufactured to sub-diffraction limit size with our donut-shaped CW visible laser system.

**Figure 3:**
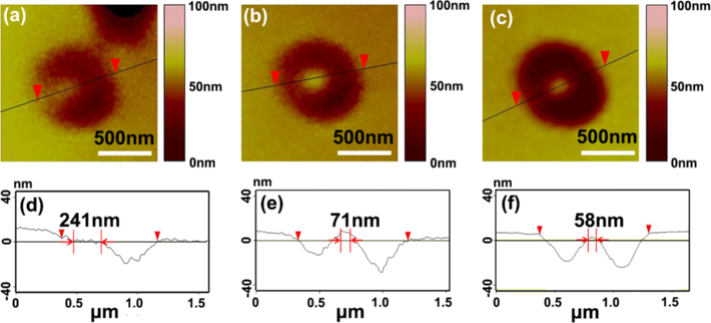
**AFM images of typical nanopillars.** (**a**) Near the rim of the pit. (**b**) Close to the center of the pit. (**c**) At the center of the pit. (**d**) Cross section of pattern in (**a**). (**e**) Cross section of pattern in (**b**). (**f**) Cross section of pattern (**c**).

Figure 3 shows the typical nanopillars fabricated in our experiments. The AFM images of Figure 3a, b, c show the three different nanopillars which are fabricated with the same laser power. Figure 3d,e,f is the corresponding cross-sectional information along the black lines in Figure 3a, b, c, respectively. These black lines are drawn as symmetry axis of the patterns in Figure 3a, b, c. It is noted that in Figure 3a, the pattern is semilunar, and the bright part, which is embedded in the semilunar pattern, has a width of about 241 nm along the dark line. The pattern in Figure 3b becomes donut-shaped, and in the pattern is the nanopillar with a pillar width of 71 nm. In Figure 3c, the nanopillar is almost located at the center of the pattern, and its pillar diameter is around 58 nm. The cross-sectional drawing (Figure 3d,e,f) reflect the asymmetry of depth in the patterns as well as the nonuniformly distributed light intensity. The depth of the left-side pit in Figure 3f is larger than that in Figure 3e, d, while the depth of the two pits in Figure 3a is the smallest. This result indicates that the focal spot has a concentrated and better symmetry of intensity distribution in the case of Figure 3c.

Comparing the experimental pillars in Figure 2 with the laser spot shown in Figure 1b, as well as in Figure 3, it seems that the nanopillars' location deviated a little from the center of the donut-shaped beam. Meanwhile, the entire donut-shaped pattern seems changed to an elliptical shape rather than a cylindrical donut shape. In order to fabricate large area-distributed nanopillar/pore array with high consistency with the system, the reasons of the nanoscale patterns transformed are systematical analyzed.

It is well known that the transformation of donut-shaped patterns might be caused by the laser quality, the photoresist surface roughness, the optical system errors, or laboratory personnel operational interferences. However, this phenomenon should not be caused by the laser beam quality because the laser focal spot has a symmetric donut shape on the focal plane which is shown in Figure 1b. Otherwise, the surface roughness should not be the issue that can be clarified in Figure 2c in which the coating photoresist surface is flat. During lithography, the laser beam is well aligned to expose the resist vertically; thus, shape deformation is not caused by a tilt photoresist wafer.

Besides the factors mentioned above, optical system errors can affect laser distribution. Spherical aberration, coma, and astigmatism are three primary factors of optical system errors. In general, the focal spot cannot be transformed to an irregular shape under the influence of spherical aberration. On the contrary, coma may cause one-directional deformation of the focal spot, while astigmatism can split the laser spot into two parts. There are two more factors: one is that this kind of laser lithography system is not sensitive to the influence of the spherical aberration; another is that the objective is designed as an aplanatic lens which eliminates the spherical aberration of the objective. Taking these factors into account, theoretical analysis and numerical calculation will be focused on the influences of coma and astigmatism effect. Aberration influence theory of the focal donut spot is described in the Appendix.

### Coma influence

**Figure 4:**
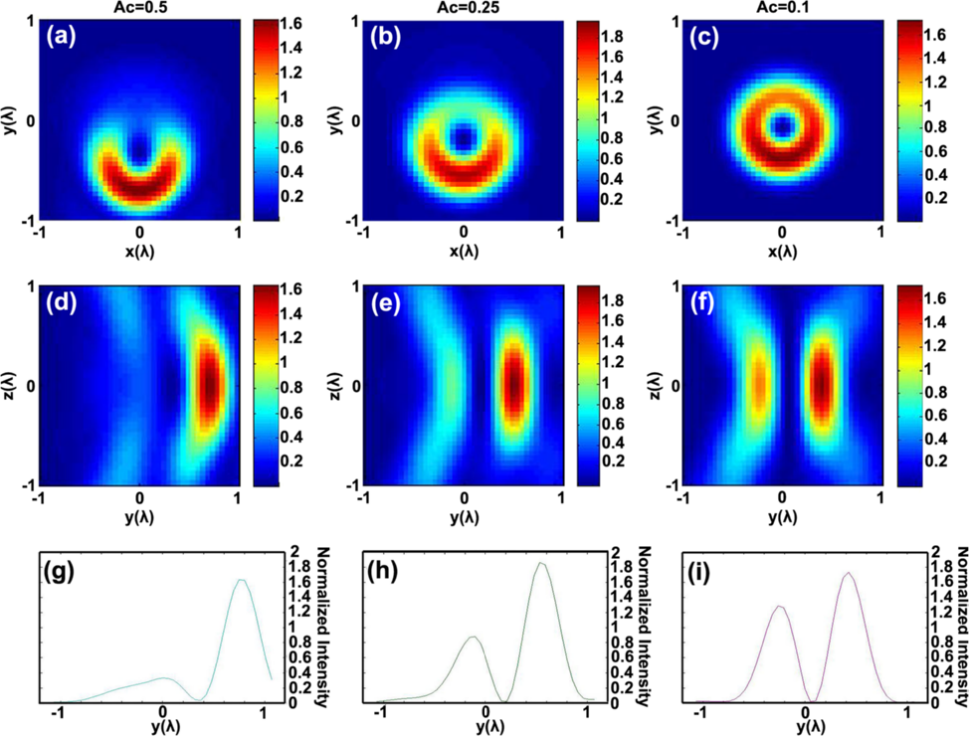
**Simulation result of coma effect.** The simulated donut-shaped focal spot intensity vs coma effect on the *xy* plane: (**a**) *A*_*c*_ = 0.5, (**b**) *A*_*c*_ = 0.25 and (**c**) *A*_*c*_ = 0.1. The corresponding intensity on the *yz* plane: (**d**) *A*_*c*_ = 0.5, (**e**) *A*_*c*_ = 0.25, and (**f**) *A*_*c*_ = 0.1. Intensity along the *y* axis: (**g**) *A*_*c*_ = 0.5, (**h**) *A*_*c*_ = 0.25, and (**i**) *A*_*c*_ = 0.1.

Figure 4 is the simulation result of coma effect for the structured laser beam as coefficient *A*_*c*_ which is assigned with different values. The intensity distribution of the donut-shaped laser spot on the *xy* plane is revealed in Figure 4a, b, c; corresponding coefficient *A*_*c*_ values are 0.5, 0.25 and 0.1, respectively. Figure 4d, e, f stands for the calculated simulations of optical intensity on the *yz* plane with *A*_*c*_ values equal to 0.5, 0.25 and 0.1 in sequence. Figure 4g, h, i shows the corresponding cross-sectional profiles of light intensity distribution on the *y* axis as *A*_*c*_ is 0.5, 0.25 and 0.1, respectively. These figures in Figure 4 clearly illustrate the gradual transformation of light distribution induced by coma effect. The dark core of the donut-shaped pattern is stretched along one direction with the increase of *A*_*c*_. Meanwhile, light intensity changes and becomes a monosymmetric distribution. It can be clearly observed that the dark spot at the core of the laser beam turns into an elliptical shape as *A*_*c*_ increases.

It makes sense to compare the results of the experiments and simulations. Their resemblances are easily found out. First, the calculated results shown in Figure 4a, b, c have similar patterns with those experimental patterns imaged in Figure 4a, b, c, respectively. The donut-shaped focal spot is a semilunar appearance in both experiment and simulation. Next, the gradual transformation of nanopillars in the experiment has the same variation tendency with the dark spots in the numerical simulation. Figure 4d, e, f illustrates the asymmetric intensity distribution on the *yz* plane; they explain the reasons why the two sides of the nanopillars are ruptured with different depths. Furthermore, Figure 4g, h, i has shown that the depletion of light intensity increased with the increased *A*_*c*_, which correctly reflects the variation of depths at the two sides of the nanopillars in Figure 4d, e, f. Thus, coma effect is the main influence factor which results in nonideal nanopillar patterns in Figures 2 and 3.

**Figure 5:**
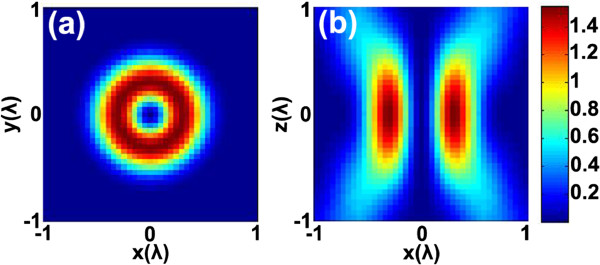
**Intensity distribution of the ideally donut-shaped laser spot.** (**a**) The lateral plane and (**b**) the vertical plane.

It should be noted that because of the conical shape of AFM probe tip, the height of the nanopillars is not exactly available with AFM observation. However, the spatial characters of the donut-shaped focal spot can be correctly reflected, and the height of the nanopillar can be relatively revealed. Figure 5 is the simulation about the donut-shaped laser distributing on the focal plane and the axial plane. It indicates that the height of the nanopillar can be as large as one λ or more.

We deem that the influence of coma effect caused by the ×100/1.4 objective lens is insignificant since this type of objective is aplanatic which dispels coma influence of the objective. Also, the focal spot has a well-defined symmetric shape before patterning the photoresist as is displayed in Figure 1b. In addition, the extents of coma effect, which is shown in Figure 3a, b, c, are different under the same experimental conditions. Therefore, we consider that coma effect of the laser lithography system should be caused by mechanical disturbance. In fact, the mechanical vibration during the system working may disturb the laser beam and then induce an angle of deviation between the laser beam and objective lens.

### Astigmatism influence

**Figure 6:**
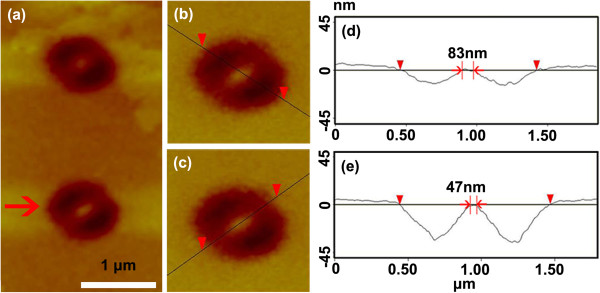
**Images of the other kind of nanopillar.** (**a**) AFM image of the other kind of nanopillar. (**b**, **c**) Enlarged image of the marked pattern in (**a**) along different directions. (**d**, **e**) The corresponding cross sections of (**b**) and (**c**).

Figure 6a presents images of the other kind of nanopillar with distorted pattern caused by astigmatism besides the situations shown in Figure 3 (the noise of background in Figure 6a is due to AFM software processing). We take the typical pattern marked by the arrow in Figure 6a. Figure 6b, c presents the zoomed-in images of the marked nanopillar in Figure 6a. In Figure 6b, c, dark lines pass through the top of the nanopillar, and they are drawn as the symmetry axes for the nanostructure in two perpendicular directions. Figure 6d, e presents the cross sections along the dark lines in Figure 6b, c, respectively. In Figure 6, it is obvious that the nanostructures fabricated by laser lithography are almost located at the center of the patterns; however, they are an elliptic cylinder. It is also evident that the patterns in Figure 6b, c,d,e are symmetric to the two dark lines, but not completely the same as that in Figure 5a. As has been explained earlier, spherical aberration influence is negligible since an aplanatic lens is employed as the objective lens. Therefore, this kind of experimental phenomenon could only be induced by astigmatism effect.

**Figure 7:**
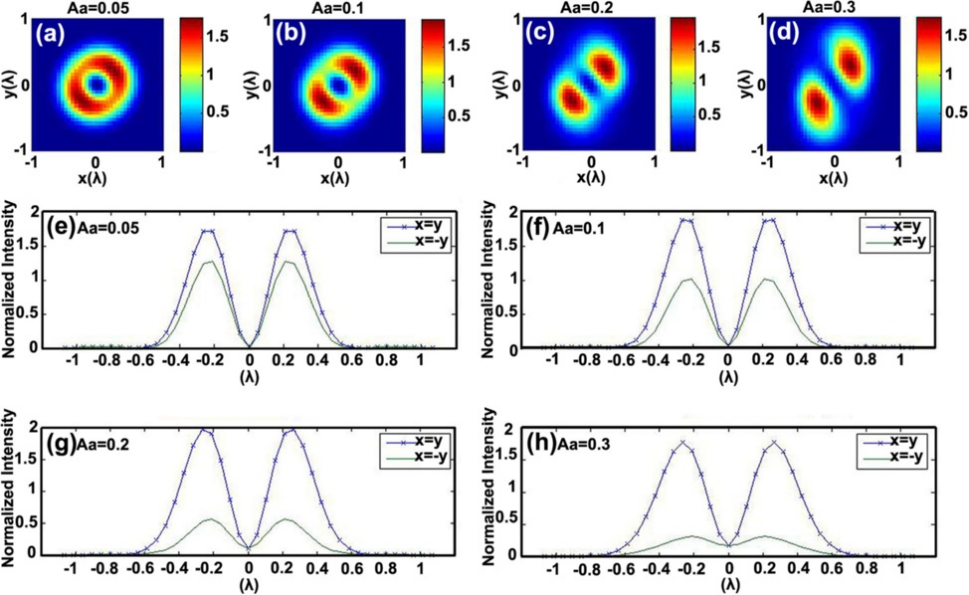
**Numerical simulation of astigmatism influence on the donut-shaped focal spot.** Simulated light intensity distribution vs astigmatism coefficient. (**a**) *A*_*a*_ = 0.05, (**b**) *A*_*a*_ = 0.1, (**c**) *A*_*a*_ = 0.2, and (**d**) *A*_*a*_ = 0.3. Intensity along *x* = *y* and *x* = −*y* (**e**) *A*_*a*_ = 0.05, (**f**) *A*_*a*_ = 0.1, (**g**) *A*_*a*_ = 0.2, and (**h**) *A*_*a*_ = 0.3.

Figure 7 is the numerical simulation of astigmatism influence on the donut-shaped focal spot. Figure 7a,b,c,d shows the intensity distribution calculated with different *A*_*a*_ values which are 0.05, 0.1, 0.2, and 0.3, respectively. The corresponding profiles of intensity distribution along *x* = *y* and *x* = −*y* are shown in Figure 7e, f, g, h. In Figure 7a, b, c,d, some clues about the gradual transformation of the donut-shaped laser spot could be found. As *A*_*a*_ is increasing, the laser pattern is pulled into two opposite directions and finally separated into two parts while the center shape varies from a circular to a belt-like structure. In addition, the light intensity in the center of the focal spot is no longer zero when *A*_*a*_ is bigger, which is apparent in Figure 7g, h.

It is also meaningful to compare the experimental results shown in Figure 6 with the simulation results in Figure 7; the pattern of the marked experimental result in Figure 6a is found very similar with the simulation result in Figure 7b with *A*_*a*_ = 0.1. It can be seen from Figure 7f that the distribution is symmetric with the origin, and the light intensity is different along *x* = *y* and *x* = −*y*. These calculated results explain the laser lithography symmetric depth on the two sides of the nanopillar shown in Figure 7d, e. The widths of the longer axis and the shorter axis of the pillar top are 83 and 47 nm, respectively, which is illustrated in Figure 7d, e.

In conclusion, combining the experimental work and the numerical simulation, it can be illustrated that the nanopillar structure could be transformed by both coma and astigmatism effects. The diameter of the nanopillar is increased and the height of the nanopillar is decreased with enhanced coma value. The shape of the nanopillar is likely to be compressed into a belt form as the astigmatism influence enhanced. In the subsequent work, the effects of coma and astigmatism of the donut-shaped laser direct writing system should be carefully dealt.

Theoretically, the resolution of this laser lithography system increases when laser intensity enhances; thus, the resolution would be extremely small. However, it cannot be that small due to optical aberration effects in the system and the material utilized in the experiment. In this work, the smallest resolution that was obtained with the photoresist OIR906 is 48 nm, which is 1/11 of the incident wavelength. It is expected that the resolution should be finer with a smaller aberration influence.

The patterning speed of the lithography system is mainly determined by factors that include the scanning speed of position stage, exposure time, and pattern complexity. In this report, it takes approximately 4 min to pattern a nanopillar array within the area of 100 × 100 μm^2^. Furthermore, an improved lithography system, which is being built in our laboratory, is capable to reduce the fabrication time to 1 min on the same pattern.

In addition, the size of the donut-shaped pattern is related to the wavelength of the incident beam. The beam with a shorter wavelength will generate a smaller donut-shaped pattern on the focal plane. Feature sizes can be tuned by shifting the wavelength of the laser with a fixed input power. In fact, we have quantitatively simulated how the donut-shaped patterns changed with the different wavelengths such as λ = 800 and 400 nm. The results showed that the radius of the pattern is 468 nm (at 800 nm) and 234 nm (at 400 nm).

## Conclusions

Nanopillar array has been successfully obtained on a spin-coated thin film of OIR906 photoresist, employing a kind of novel visible CW laser direct lithography system. The diameter of the fabricated nanopillar was able to be as small as 48 nm, which is 1/11 of the wavelength of the incident laser. The lithographic nanopatterns were calibrated and analyzed with AFM. Shape influences of the coma effect and astigmatism effect were simultaneously analyzed using vector integral. The simulation results explain the distortion and inconsistency of the fabricated nanopatterns well. The work has demonstrated a simple, efficient, and low-cost method of fabricating nanopillars. It could pave a new way to fabricate nanopillars/pore arrays of large area distribution for optical nanoelements and biophotonic sensors while integrated with high-speed scanning system.

## Appendix

### Aberration theory about high NA objective

**Figure 8:**
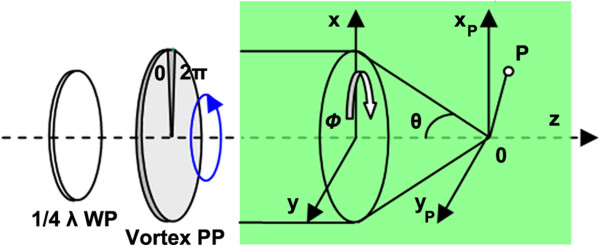
Schematic drawing of light intensity distribution on the focal plane.

Figure 8 is a schematic for laser spot distribution on a focal plane. The Gaussian beam is converted clockwise, is polarized by WP, and then passes through the PP and incident into the high NA objective lens. The components of the diffracted electric field at point *P*, which is near to the focal spot, can be expressed by the vectorial Debye theory as in Equation 1 [32]:

(1) $$\begin{array}{l} E\left(p\right)=\left(\begin{array}{c} E_{x}\\ E_{y}\\ E_{z} \end{array}\right)=-\frac{\mathrm{if}l_{0}}{\lambda }\int_{0}^{\alpha }\int_{0}^{2\pi }E_{0}\sqrt{\cos\theta }A_{1}\left(\theta ,\phi \right)\exp\\ \;\left[\mathrm{ik}\left(x\sin\theta \cos\phi +y\sin\theta \sin\phi +z\cos\theta \right)\right]\\ \times \varphi_{s}\left(\theta ,\phi \right)\left[\begin{array}{c} \cos\theta \cos^{2}\phi +\sin^{2}\phi -i\sin\phi \cos\phi \left(\cos\theta -1\right)\\ \cos\phi \sin\phi \left(\cos\theta -1\right)-i(\cos\theta \sin^{2}\phi +\cos^{2}\phi )\\ -\sin\theta \left(\cos\theta -i\sin\phi \right) \end{array}\right]\\ \;\sin\mathrm{\theta d\theta d\phi} \end{array}$$

where *f* is the focal length of the lens and *l*_0_ represents the amplitude factor in the image space; *E*_0_ is the amplitude of input Gaussian beam; *A*_1_(*θ*, *ϕ*) is the wavefront aberration function, *θ* is the angle between the optical axis and given ray; *ϕ* is the azimuthal coordinate at the input plane and *φ*_*s*_(*θ*, *ϕ*) is the phase delay generated by the phase mask; *x*, *y*, and *z* indicate the Cartesian coordinates of the point *p* in the focal region; *i* is the plural; *k =* 2*πn*/*λ* stands for the wave number, where *λ* is the wavelength of the incident light and *n* is the refractive index of the focal space medium.

The amplitude of the Gaussian beam at the input plane is expressed as in Equation 2:

(2) $$E_{0}\left(\gamma ,\theta \right)=A_{0}\exp\left(-\gamma^{2}\rho^{2}\right)$$

where *A*_0_ is the amplitude, *γ* is the truncation parameter and expressed as *γ = a*/*ω* (*a* is the aperture radius and *ω* is the beam size at the waist), while *ρ* stands for the radial distance of a point from its center normalized by the aperture radius of the focusing system and *ρ =* sin*θ*/sin*θ*_max_, where *θ*_max_ is the maximal semi-aperture angle of the objective lens, and in our system, *θ*_max_ = 67.07°.

*A*_1_(*θ*, *ϕ*) represents wavefront aberration as expressed as in Equations 3 and 4:

Coma:

(3) $$A_{1}\left(\theta ,\phi \right)=\exp\left[\mathrm{ik}A_{c}\lambda {\left(\frac{\sin\theta }{\sin\theta_{\max}}\right)}^{3}\cos\phi \right]$$

Astigmatism:

(4) $$A_{1}\left(\theta ,\phi \right)=\exp\left[\mathrm{ik}A_{a}\lambda {\left(\frac{\sin\theta }{\sin\alpha }\right)}^{2}\cos^{2}\phi \right]$$

*A*_*c*_ and *A*_*a*_ are coefficients for coma and astigmatism, respectively. Both *A*_*c*_ and *A*_*a*_ multiply *λ*, representing the departure of the wavefront at the periphery of the exit pupil. The values for *λ*, *n*, NA and *θ*_max_ adopted in simulation correspond to the practical values in the experiment. Refractive index of oil *n* = 1.52; *γ* is supposed to be 1, which means that the objective is fulfilled by the Gaussian beam.

Therefore, the intensity distribution at point *P* is written as in Equation 5:

(5) $$I\left(p\right)={\left|E_{x}\right|}^{2}+{\left|E_{y}\right|}^{2}+{\left|E_{z}\right|}^{2}$$

The electrical distributions for the donut-shaped pattern affected by aberrations are carried out using Matlab software.

## Abbreviations

AFM: Atomic force microscopy; CCD: Charge-coupled device; CW: Continuous wave; NA: Numerical aperture; PP: Phase plate; WP: Wave plate

## Competing interests

The authors declare that they have no competing interests.

## Authors' contributions

CZ carried out specimen preparation, data acquisition and analysis of measurement and simulation and drafted the manuscript. KW, JB, GW and CG conceived the experiment, designed the plan and directed the drafting of the manuscript. SW, WZ and FY contributed to the simulation program improvement and participated in drafting the manuscript. All authors read and approved the final manuscript.

## Authors' information

CZ is a Ph.D. candidate of the Institute of Photonics and Photo-technology, Northwest University, Xi'an, China, with a research direction that is concerned on laser technology and application. KW is a professor of the Institute of Photonics and Photo-technology, Northwest University, Xi'an, China. His research direction focuses on nanotechnology, nanobiophotonics, and soft matter physics. JB is a professor of the Institute of Photonics and Photo-technology, Northwest University, Xi'an, China. His main research areas are all-solid-state laser, laser devices and laser technology. SW is a lecturer of the Institute of Photonics and Photo-technology, Northwest University, Xi'an, China. His study concentrates on biophotonics and biomedical optics. WZ is a Ph.D. candidate of the Department of Mechanical Engineering, University of South Carolina, Columbia, USA. His research topics are related to applied optics and fluid dynamics. FY is a postdoc in the Department of Mechanical Engineering, University of South Carolina, Columbia, USA. He works on high resolution microscopy system and MEMS. CG is a researcher of Institute of Physics, Chinese Academy of Sciences, Beijing, China. He works in the fields of nanostructure and nanodevices. GW is an associate professor at the Department of Mechanical Engineering and is interested in nanotechnology, bioMEMS, and lab-on-chip.
